# Advances in Wastewater-Based Epidemiology for Pandemic Surveillance: Methodological Frameworks and Future Perspectives

**DOI:** 10.3390/microorganisms13051169

**Published:** 2025-05-21

**Authors:** Weihe Zhu, Daxi Wang, Pengsong Li, Haohao Deng, Ziqing Deng

**Affiliations:** 1 Beijing Key Laboratory for Source Control Technology of Water Pollution, College of Environmental Science and Engineering, Beijing Forestry University, Beijing 100083, China; 2 Hebei Key Laboratory for Emerging Contaminants Control and Risk Management, College of Environmental Science and Engineering, Beijing Forestry University, Beijing 100083, China; 3 Engineering Research Center for Water Pollution Source Control & Eco-remediation, College of Environmental Science and Engineering, Beijing Forestry University, Beijing 100083, China; 4 BGI Research, Beijing 102601, China

**Keywords:** wastewater-based epidemiology, public health surveillance, sampling, pathogen detection, epidemic forecast modeling

## Abstract

Wastewater-based epidemiology (WBE) has emerged as a transformative approach for community-level health monitoring, particularly during the COVID-19 pandemic. This review critically examines the methodological framework of WBE systems through the following three core components: (1) sampling strategies that address spatial–temporal variability in wastewater systems, (2) comparative performance of different platforms in pathogen detection, and (3) predictive modeling integrating machine learning approaches. We systematically analyze how these components collectively overcome the limitations of conventional surveillance methods through early outbreak detection, asymptomatic case identification, and population-level trend monitoring. While highlighting technical breakthroughs in viral concentration methods and variant tracking through sequencing, the review also identifies persistent challenges, including data standardization, cost-effectiveness concerns in resource-limited settings, and ethical considerations in public health surveillance. Drawing insights from global implementation cases, we propose recommendations for optimizing each operational phase and discuss emerging applications beyond pandemic response. This review highlights WBE as an indispensable tool for modern public health, whose methodological refinements and cross-disciplinary integration are critical for transforming pandemic surveillance from reactive containment to proactive population health management.

## 1. Introduction

In the face of rapid population growth, urbanization, and the persistent threat of emerging infectious diseases, effective epidemic surveillance has become a public health priority [1,2,3]. Traditional monitoring methods, including epidemiological surveys and laboratory testing, face challenges such as sampling bias, time delays, high costs, and ethical concerns. Furthermore, these methods struggle to provide real-time insights into the critical timing and locations of epidemic outbreaks [4,5]. The COVID-19 pandemic highlighted the limitations of these conventional approaches, sparking interest in wastewater-based epidemiology (WBE) as a complementary tool for rapid community-level monitoring.

During the COVID-19 pandemic, WBE proved especially valuable as a quick and non-invasive method for detecting viral pathogens in wastewater before clinical symptoms appear, including asymptomatic cases that would otherwise go unnoticed [6,7,8]. This approach leverages viruses excreted by infected individuals, allowing public health officials to estimate the overall health status of a population by analyzing the viral load in wastewater samples [8,9].

The practice of detecting viral pathogens in wastewater dates back to the 1960s, when wastewater surveillance for poliovirus emerged as a pivotal strategy in global poliomyelitis eradication campaigns [10,11,12]. These pioneering efforts fundamentally established the analytical framework of WBE. In 1999, researchers proposed systematic wastewater monitoring as an innovative approach for estimating illicit drug consumption patterns at the population level [13]. Subsequent advancements in 2005 demonstrated the methodology’s expanded potential through the successful quantification of cocaine metabolites in municipal wastewater systems [14]. Over the past two decades, WBE has developed into a multidisciplinary surveillance paradigm, with contemporary applications spanning pharmaceutical monitoring, environmental contaminant tracking, and increasingly, public health surveillance [14,15,16,17].

In addition, WBE has played an important role in the global polio eradication initiative by continuously monitoring updates on vaccination coverage and predicting public health actions before epidemic outbreaks [18]. Since the outbreak of COVID-19 in 2020, WBE has rapidly gained popularity due to its non-invasive, fast, and convenient nature, and it has been applied to other areas, such as monitoring the interactions of common respiratory viruses and the transmission of different influenza virus subtypes [19,20,21].

The early phase of WBE primarily focused on monitoring drug metabolites, relying on liquid chromatography-mass spectrometry (LC-MS) for analysis. Although Italy first detected cocaine consumption in wastewater in 2005, detection sensitivity at the time was relatively low (detection limit > 50 ng/L) [14].

Advances in pathogen detection technologies, particularly quantitative polymerase chain reaction (qPCR) and metagenomics, improved the sensitivity and specificity of WBE for detecting a wider range of pathogens in wastewater. However, the absence of standardized methods led to significant prediction errors, with sampling techniques alone introducing errors of up to 10% [22].

In recent years, AI-driven early warning systems and global collaborative networks have become central to WBE. The establishment of the WHO WBE database has been key in promoting cross-national data sharing and integration. Notably, in 2020, the Netherlands used machine learning models to predict COVID-19 outbreaks seven days earlier than expected [23], highlighting the increasing importance of WBE in public health surveillance.

**Figure 1:**
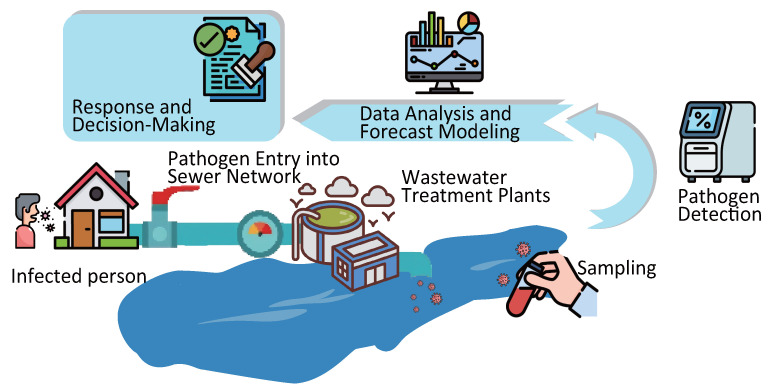
Diagram of the WBE workflow.

WBE enables real-time tracking of viral transmission dynamics and offers valuable insights into the efficacy of public health interventions. The core process involves sampling, pathogen detection, data analysis, and forecast modeling, guiding the responses and decision making regarding the pandemic (Figure 1) [13,24,25,26]. By detecting changes in the types, concentrations, and flux of viruses in wastewater, WBE helps predict epidemic outbreaks and guide targeted interventions.

However, realizing WBE’s full potential involves overcoming several challenges. As their survival and behavior in sewage systems vary, not all pathogens are easily detected in wastewater, which poses a significant barrier [27,28]. Therefore, sampling and detection techniques require refinement to improve accuracy, reduce costs, and enhance predictive capabilities [15]. Additionally, the precision of the current predictive models is often constrained by data accuracy and the complexity of environmental variables [29].

In this review, nearly 200 representative studies in the field of WBE are examined and categorized according to WBE steps. Ultimately, this review provides a comprehensive examination of the main steps in the WBE process, from sample collection to data analysis and predictive modeling. By comparing case studies and analyzing the latest technological advances, the article identifies key challenges and opportunities for WBE, offering recommendations for optimizing its application in future public health efforts. Although the determination of drugs in wastewater is also based on the concept of WBE, in terms of epidemiology itself, WBE focuses more on monitoring pathogens, especially viral pathogens, in wastewater [18]. Therefore, we focus on viral pathogens in this review.

## 2. Sampling in WBE

The accuracy of WBE relies heavily on systematic sampling and the sample pre-treatment methods [30], as the quality of primary data obtained from sampling directly determines the validity of subsequent analyses [31]. Establishing reliable sampling strategies requires understanding the environmental persistence of viral nucleic acids. Key determinants of viral nucleic acid stability in water systems are temporal and spatial factors, with water temperature emerging as the most critical parameter [7,32,33,34]. Experimental evidence shows SARS-CoV-2 RNA exhibits differential persistence across environments as follows: in wastewater and tap water at room temperature, the time for the 90% reduction ($T_{90}$) of viable virus is 1.5 and 1.7 days, respectively, [34]. Notably, under high initial titers, infectious virus can persist in pipelines for up to 7 days, and heating can effectively reduce $T_{90}$ [34]. Similar patterns emerge in natural waters, where infectious SARS-CoV-2 persists for 2.3 days in river water and 1.1 days in seawater at 20 °C [35]. These spatiotemporal patterns of viral persistence fundamentally inform the design of sampling strategies.

WBE sampling strategies are typically defined by four key factors as follows: location, timing, frequency, and method. By carefully designing a sampling strategy, WBE provides critical insights into where epidemic outbreaks may begin and how they spread, helping public health officials allocate medical resources more effectively, particularly in large, resource-limited regions.

### 2.1. Sampling Locations

Selecting the right sampling sites is crucial for accurate data collection. Researchers often use geographic information system (GIS) data to analyze urban landscapes and ensure representative sampling across different terrains and regions [36]. In addition to GIS data, it is important to consider the characteristics of sewage networks, including the layout of pipelines, pipe sizes, flow rates, and topography [37]. High-flow pipelines or wastewater treatment plants in densely populated areas are often preferred as they reflect the health status of larger populations [37,38]. For instance, WBE studies commonly select wastewater treatment plants in major urban centers to track viral trends and predict potential outbreaks [23,39].

### 2.2. Sampling Timing

Sample timing plays a critical role in WBE accuracy. Wastewater discharge can fluctuate due to daily activities and seasonal changes, affecting viral concentrations [40]. Generally, sample collection should avoid peak discharge periods to minimize variability [30]. However, during epidemic outbreaks, sampling at peak times may be necessary to capture the maximal viral loads [39,41]. Seasonal factors, such as holidays or tourist seasons, also impact wastewater composition and should be accounted for when planning the sampling schedule [23,42,43,44]. Timely analysis after sampling is critical, as viral nucleic acids degrade rapidly, with even minor delays potentially compromising data integrity [43].

### 2.3. Sampling Frequency

Sampling frequency is another critical factor in WBE. While higher sampling frequencies provide more granular data [30,38], they may not always be the most effective, especially for pathogens with low-frequency signals [37]. In some cases, less frequent, strategically timed sampling yields more reliable results [31]. The optimal sampling frequency often depends on the incubation periods of specific pathogens, with shorter incubation periods requiring more frequent sampling to capture transmission trends accurately [38]. Detection technologies such as quantitative polymerase chain reaction (qPCR) and sequencing have specific sampling frequency needs, which must be integrated into the overall strategy [45].

### 2.4. Sampling Methods

The following two methods are commonly used to collect wastewater samples: composite sampling and grab sampling. Composite sampling, which collects samples over a set time, is more reliable, as it accounts for fluctuations in wastewater flow and composition, reducing the risk of bias from a single time point. Grab sampling, by contrast, provides a snapshot of conditions at a specific moment, which may not accurately represent broader trends [46]. Among composite sampling techniques, Kitamura et al. [47] considers solid precipitation to be the most ideal approach. Meanwhile, Babler et al. [48], by evaluating multiple microbial indicators in 24-h mixed wastewater samples, concluded that significant hourly variations in SARS-CoV-2 abundance could affect the representativeness of mixed samples for daily averages. Therefore, to preserve the integrity of samples, refrigeration or the use of ice during transport is recommended, especially in warmer climates where viral degradation is more likely [34,48,49,50]. Furthermore, factors such as water quality and wastewater retention time in pipelines should be considered, as they can affect the stability of viral RNA [51]. Finally, the sampling process also needs to be adjusted based on the specific requirements of the locations [31]. For instance, in underdeveloped or remote regions without established sewage systems, as well as in schools with rotating schedules, where establishing a connection between viral load and cases may be challenging, it is necessary to update the sampling frequency [52].

## 3. Pathogen Detection Techniques in WBE

WBE employs several techniques to detect viral genetic material, with the most common methods being qPCR, sequencing, and biosensors. Each of these methods offers unique advantages and limitations, which must be considered depending on the specific goals of the study (Table 1).

### 3.1. qPCR: The Gold Standard for Pathogen Detection

qPCR is the most widely used method for detecting viral RNA in wastewater. It converts viral RNA to DNA and amplifies specific genomic regions, allowing for highly sensitive and specific detection [19].

To enhance detection accuracy, researchers often concentrate virus particles using precipitation methods (such as polyethylene glycol) or physical techniques (like ultracentrifugation and ultrafiltration) before applying qPCR [53,54].

The adsorption-extraction method is particularly efficient and commonly used. For example, in the case of COVID-19, qPCR-based approaches typically target the N1 and N2 regions of the viral genome [49]. However, variants of concern (VOCs) with mutations in regions like the S gene (which encodes the spike protein) can lead to diagnostic failures, as seen with variants like Omicron [55]. Additionally, the complex matrix of wastewater can degrade viral nucleic acids or introduce inhibitors, limiting the effectiveness of PCR [56].

Despite these challenges, qPCR remains the cornerstone of WBE due to its multiple advantages. It allows for multiplex detection, meaning that multiple viral targets can be identified in a single assay, which is particularly useful in low-concentration samples [57,58]. Additionally, qPCR provides rapid results (approximately 4.5 h), whereas next-generation sequencing (NGS) can take 48–60 h due to the need for enrichment steps.

To overcome qPCR’s limitations, researchers have developed alternative approaches. Droplet digital PCR (ddPCR), for example, partitions samples into thousands of droplets, allowing for absolute quantification and reducing the impact of variable amplification efficiency [59,60]. This method provides higher sensitivity and lower detection limits compared to traditional qPCR [61,62]. Other techniques, such as microbial source tracking (MST) combined with qPCR, have been employed to trace fecal contamination, using CrAss-like phages as biomarkers to assess viral prevalence in wastewater [63].

### 3.2. Sequencing: Beyond the Gold Standard

Sequencing technologies have become essential tools in WBE, providing a significant advantage over qPCR by offering detailed nucleic acid sequence information [49,64,65]. This capability enables for the differentiation of various pathogens and their subtypes, facilitating large-scale sample processing and improving the surveillance of population-level disease dynamics [23,66,67,68,69]. Sequencing technologies also lead to breakthroughs in identifying co-infections, understanding the ecological roles of viruses, and monitoring the impact of environmental changes on microbial communities [70,71,72]. Amplicon sequencing and metagenomic sequencing are the two main sequencing strategies used in WBE, each with distinct applications and benefits.

#### 3.2.1. Amplicon Sequencing

Amplicon sequencing is widely used in WBE due to its cost-effectiveness and relatively simpler analytical methods. This technique focuses on the composition and diversity of species, allowing for the detection and identification of known pathogens in wastewater [73]. Its maturity and widespread use in WBE is valuable for large-scale surveillance [74,75,76]. However, since amplicon sequencing relies on pre-designed primers for known pathogens, its ability to detect unknown or emerging pathogens is limited [77]. Despite this, amplicon sequencing remains a cornerstone in WBE, particularly for studies requiring frequent, cost-efficient monitoring of specific viruses or microbial communities.

#### 3.2.2. Metagenomic Sequencing

In contrast to amplicon sequencing, metagenomic sequencing provides a more comprehensive view of all genetic material present in a sample, not just pre-selected regions [78]. This unbiased approach enables the detection of a wide array of pathogens, including unknown or unexpected ones, making it valuable for discovering new pathogens or variants in wastewater [79].

The broader scope of metagenomic sequencing provides deeper insights into the microbial diversity in wastewater, making it possible to study co-infections and interactions between different microorganisms [78]. However, the extensive data produced by metagenomic sequencing require advanced bioinformatics tools, and the higher costs of this approach limit its application in WBE [70,71,80]. Despite these limitations, as technology advances and costs decrease, metagenomic sequencing is poised to play a crucial role in the early detection of emerging pathogens and in providing a more holistic understanding of wastewater microbiomes [17,76,81].

#### 3.2.3. Sequencing Platforms: Short-Read vs. Long-Read

The choice of sequencing platform can significantly impact the outcomes of WBE studies. Short-read sequencing, which utilizes high-throughput sequencing (HTS) platforms such as Illumina and MGI, is the most commonly used due to its lower cost and established protocols. These platforms are highly effective for detecting known pathogens and estimating viral loads, making them widely used in routine WBE applications. However, short-read sequencing has limitations when reconstructing complex viral genomes, as the short fragments can make it difficult to detect large-scale structural variations [82].

In recent years, long-read sequencing platforms like PacBio, Nanopore, and CycloneSEQ have been gaining attention due to their ability to sequence much longer DNA or RNA fragments, providing near-complete genome coverage [83]. This capability not only minimizes biases introduced by sequence assembly, thereby enhancing the accuracy of viral genome reconstruction, but it also enables the detection of large-scale structural variations and facilitates the reconstruction of complete genomes. Long-read sequencing is particularly valuable in situations where the viral genomes are complex and highly fragmented, as often occurs in wastewater samples [83,84,85]. Platforms like Nanopore and CycloneSEQ allow for real-time data collection, facilitating more rapid responses to changes in viral populations in wastewater.

Although long-read sequencing demands high-quality nucleic acids, advancements in library preparation methods, including protocols designed to handle degraded samples, are progressively mitigating these issues [75,86]. Techniques such as removing inhibitors, optimizing DNA extraction, employing nucleic acid repair, using enhanced library preparation reagents, and standardizing fragmentation steps are contributing to the more effective use of long-read sequencing in wastewater samples. While further validation is needed for samples with low viral loads, the unique capacities of long-read sequencing make it a powerful tool for comprehensive genomic analysis in WBE [71].

#### 3.2.4. Challenges of Sequencing in WBE

While sequencing technologies offer unparalleled insights into viral diversity and dynamics, they also present challenges such as variability in sample extraction efficiency, time-consuming library preparation, and high costs, especially for metagenomic sequencing [78]. Additionally, the interpretation of sequencing data requires sophisticated bioinformatics tools and expertise, posing a barrier to the application of sequencing technologies in WBE [71]. Despite these challenges, sequencing technologies continue to advance, and their unique capabilities make them invaluable tools for comprehensive genomic analysis in WBE.

### 3.3. Biosensors: Emerging Tools for Flexible Detection

In addition to genetic material analysis methods like qPCR and sequencing, biosensors have emerged as flexible and innovative tools for detecting viral markers in wastewater. Biosensors employ specific receptors to detect target substances (e.g., viral proteins or nucleic acids) via biochemical reactions that generate measurable optical or chemical signals [87,88,89].

#### 3.3.1. Types of Biosensors in WBE

Biosensors in WBE can be broadly categorized into the following two types based on the need for amplification: amplification-based and non-amplification biosensors.

Amplification-based biosensors rely on amplification techniques to detect specific molecules, such as viral RNA. For instance, reverse transcription loop-mediated isothermal amplification (RT-LAMP) can amplify viral RNA to detectable levels [90]. Ahn et al. [91] designed a RT-LAMP-based biosensor for rapid diagnosis of the Zika virus, and similar approaches have been adapted for SARS-CoV-2 [92]. In addition, Yang et al. developed an origami microfluidic sentinel sensor based on the same principle [93,94]. This biosensor offers the advantages of easy signal reading via mobile phones and low cost, making it particularly suitable for use in low- and middle-income countries and resource-limited areas. Furthermore, Torres-Salvador et al. [89] proposed an electrochemical biosensor (E-Biosensor) that employs isothermal nucleic acid sequence-based amplification (NASBA) to detect multiple viruses through hybridization with specific probes.

Non-amplification biosensors detect viral markers through optical, electrochemical, or thermal signals without the need for amplification. For example, optical biosensors can detect viral proteins, while electrochemical sensors can measure viral RNA directly. One of the most promising non-amplification biosensors is based on field-effect transistors (FET). Seo et al. [95] developed a COVID-19 detection method using FET biosensors, which detects viral antigens in clinical samples with high sensitivity and rapid detection speed [96,97]. Although these biosensors offer significant advantages, including the low risk of sample contamination, their application in wastewater is limited by the need for specific sample types and conditions.

#### 3.3.2. Applications of Biosensors in WBE

Biosensors have shown great promise in WBE for inferring population health and public health conditions in specific regions. They offer several key advantages over traditional detection methods as follows: they are non-invasive, can be used on-site for continuous monitoring, and are capable of delivering results quickly. These characteristics make them especially useful in detecting dynamic changes in viral concentrations in wastewater, which are particularly valuable for WBE [98]. Their relatively low cost and ease of use allow biosensors to be deployed in resource-limited areas, where traditional laboratory methods may not be feasible and rapid, real-time monitoring is needed [99,100].

In WBE, biosensors have already been used to detect viral RNA and proteins, heavy metal ions, and other chemical markers that provide insights into public health and disease trends [99,100,101,102,103]. Beyond viral load detection, biosensors can also track host responses to infections, such as immune functions and inflammatory processes [102,103,104,105], offering a comprehensive view of public health.

#### 3.3.3. Emerging Innovations of Biosensors in WBE

Biosensors are rapidly evolving in WBE. Innovations, such as those integrating biosensors with mobile health (mHealth) platforms or combining them with microfluidics for point-of-care diagnostics, are being explored [102]. These advancements will allow biosensors to monitor population health more efficiently and provide real-time data on epidemic outbreaks. Additionally, mobile-based biosensors could expand access to monitoring, especially in underdeveloped regions, offering a more scalable and affordable solution for public health surveillance.

Efforts are ongoing to improve the sensitivity and specificity of biosensors, making them capable of detecting lower concentrations of viral particles in wastewater [98]. This would increase their utility in the early detection of outbreaks, even at low viral prevalence [103]. For instance, integrating biosensors with digital health technologies could allow the real-time transmission of data to centralized public health systems, offering a faster, more coordinated response to potential outbreaks [102].

#### 3.3.4. Challenges of Biosensors in WBE

Despite their potential, biosensors face several limitations in WBE. Amplification-based biosensors may introduce errors during amplification, while non-amplification biosensors often require specific environmental conditions or sample types to function effectively [98]. Additionally, biosensors may struggle with sample contamination or degradation in wastewater systems, which can affect the reliability of the results.

Another challenge is ensuring the reproducibility and standardization of biosensor data, especially when applied across different regions with varying wastewater compositions [99,100,101,102,103]. There are also technical limitations associated with scaling biosensor technology for large populations. While high-throughput testing with biosensors is possible, it remains undeveloped compared to the widespread use of qPCR and sequencing methods in WBE [106]. Overcoming these challenges requires the further technological innovation and validation of biosensor systems in real-world wastewater monitoring scenarios.

## 4. Data Preprocessing and Epidemic Forecast Modeling

### 4.1. Data Preprocessing

Once samples are collected and analyzed using advanced detection methods—qPCR for viral identification, or sequencing and biosensors for comprehensive microbial profiling—the raw data undergo critical preprocessing. This step ensures that the datasets are refined and normalized, setting a solid foundation for accurate and meaningful analysis in WBE [107].

Effective data preprocessing is the foundation of WBE, enabling the accurate interpretation of viral signals in wastewater and predicting epidemic outbreaks. Comprehensive data preprocessing is essential to ensure accuracy and reliability before any data analysis can begin [108]. This process includes data normalization, evaluation, and adjustment.

#### 4.1.1. Data Normalization

Data normalization is the first and most crucial step for ensuring data accuracy. Data normalization mainly consists of the following two parts: data cleaning, and data transformation and standardization [109].

In WBE, data cleaning is crucial because viral signals degrade over time. Confounding variables (such as chemical pollution, weather, and human activities) introduce noise [48]. Therefore, it is necessary to detect anomalies and handle missing data.

After cleaning the data, transformation and standardization are performed. Smoothing algorithms are used to highlight trends, particularly in the context of extreme weather events. Data are segmented based on specific events and indicators to accurately reflect different patterns of viral concentration. Additionally, relevant background variables (e.g., environmental and sociocultural factors) are converted into vectors and incorporated [48,110].

#### 4.1.2. Data Evaluation and Adjustment

After normalization, it is necessary to select important features and divide the data into appropriate datasets. Dimensionality reduction is conducted to select features that are strongly correlated [110]. Then, the preprocessed data are divided into training and testing sets according to needs, typically using an 80–20 split [110]. In addition, external factors such as extreme weather, industrial emissions, and changes in population behavior are considered to reevaluate data reliability [34,48,49,50].

Parallel control or probabilistic models are used to compensate for these effects, to ensure the robustness of predictions. External data (such as meteorological and demographic information) are integrated into the analysis, and models are regularly updated with new data to enhance the accuracy of long-term monitoring [110].

### 4.2. Epidemic Forecast Modeling

A solid foundation of thorough data preprocessing ensures the reliability and consistency of input data, laying the necessary groundwork for effective predictive analytics. Predicting epidemic outbreaks from wastewater data require advanced data modeling techniques, which play a crucial role in integrating diverse data sources and forecasting trends [29]. Machine learning models, in particular, can process large datasets and generate accurate predictions using real-time data, which is essential for forecasting epidemic outbreaks, assessing trends, and informing public health policies [111,112,113].

The modeling process typically begins by feeding evaluated data—such as virus concentrations, population, pipeline characteristics, and hydrological information—into machine learning algorithms. These models are supervised and validated by experts to generate predictions that can inform government policies and intervention strategies [114].

**Figure 2:**
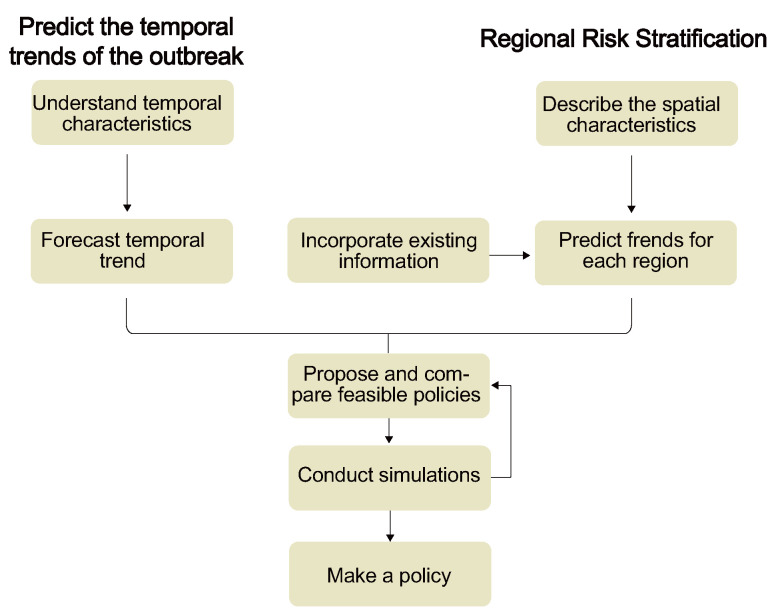
Flow chart of epidemic forecast modeling.

The core challenges addressed by WBE can be categorized into the following two types: predicting the temporal trends of an epidemic and regional risk stratification (Figure 2) [30,37,38,39,41,108,115,116,117,118].

The former relates to time-based problems and thus requires time series analysis, while the latter pertains to spatial issues and necessitates spatial forecasting analysis.

#### 4.2.1. Modeling for Predicting the Temporal Trends of Epidemics

To predict the temporal trends of epidemics, it is essential to first apply descriptive models to understand the data structure and temporal characteristics, laying the groundwork for predictive and normative modeling [115,119,120]. Since the focus is on overall trends, the presence of numerous epidemic-related features can cause to the problem of overfitting in high-dimensional data, thereby compromising model stability [114]. Therefore, dimensionality reduction should be conducted first for the better analysis of the main data structure and to eliminate unnecessary data. Next, feature importance should be analyzed.

Subsequently, clustering analysis, such as K-means and hierarchical clustering, can be employed to identify data groupings and assess inter-sample similarities [121,122,123]. Ultimately, key features are extracted, often including “transmission characteristics” (e.g., infection rates) and “impact characteristics” (e.g., severe case rates and mortality rates). A descriptive model can then be developed to quantitatively describe the current temporal characteristics of the epidemic, such as peak periods, transmission rates, and periodic fluctuations [119].

Based on insights from the descriptive model, predictive models should be developed to forecast future trends and support resource planning [118]. Predictive models can be classified based on their output type into regression models and classification models [118]. Given that epidemic trends over time are a continuous variable, temporal regression models are preferred. When selecting regression models, it is important to select the appropriate ones based on the size of the dataset.

For smaller datasets, simpler regression models such as linear regression and nonlinear regression are commonly used [124,125,126]. The former is suitable for linear relationships and is preferred when strong interpretability and high computational efficiency are needed; polynomial regression is a representative example [126,127]. The latter can fit more complex nonlinear relationships, with Support Vector Regression (SVR) being particularly effective when focusing on VOCs [30]. However, prolonged use can lead to inefficiencies due to high computational complexity.

For larger datasets, algorithms like Artificial Neural Networks (ANNs) and Random Forests (RFs) are typically employed. For instance, Jiang et al. [116] used an ANN to estimate the effective reproduction rate $R_{i}$ of the virus based on wastewater samples and other known information from 47 wastewater treatment plants in Utah, USA, while Vaughan et al. [38] successfully used a RF regression model to predict weekly hospitalizations during the COVID-19 pandemic.

Once the predictive results are obtained, additional known data —such as historical epidemic records, social behavior characteristics, and environmental factors—can be incorporated to optimize the model. The resulting data can be used for policy evaluation [128].

#### 4.2.2. Modeling for Regional Risk Stratification

The second type of problem regarding regional risk stratification focuses on identifying important features associated with spatial factors [129]. Descriptive models are also used to describe the current spatial characteristics of the current situation. Region-specific regression models predict epidemic trends while accounting for inter-regional interactions [130]. After establishing the initial regression models, additional information is incorporated, often with assistance from GIS [128]. Classification models are a form of supervised learning that can predict categories with specific labels. Based on the optimized models, the classification objectives are first defined, segmenting different time periods and designing labels and thresholds [131,132,133]. Bayesian and RFs classification models are usually used at this stage. For instance, Rallapalli et al. [134] employed a Bayesian model to identify high-risk areas during the pandemic and to assess the effectiveness of vaccine distribution, while Li et al. [118] predicted weekly new hospitalization cases in 150 county-level regions in the United States based on an RF model, demonstrating these models’ ability to classify data based on regional conditions [135].

#### 4.2.3. Machine Learning Models Used in WBE

As previously discussed, machine learning models can enhance WBE as a monitoring tool. When applied effectively, they can transform WBE from a passive monitoring approach into an active epidemic prevention system. By assessing viral trends and predicting epidemic outbreaks in real time, these models provide critical insights for policymakers [36,136].

Each machine learning model presents unique advantages that are contingent upon the specific context (Table 2). For instance, Polynomial regression and SVR excel at detecting complex patterns in viral data, but they require significant computational resources. RF models, in contrast, are versatile and can handle complex datasets with multiple variables and missing data, making them ideal for large-scale, real-world applications. Support vector machines (SVMs) are well suited for short-term forecasting and identifying the impact of pipeline systems on virus spread [137], as demonstrated by Zehnder et al. [37].

Regarding feature selection, the Simple Moving Average (SMA) and Locally Weighted Regression (LOESS) are commonly used. SMA is a robust and easy-to-implement method, particularly suitable for smoothing simple short-term data [30]. In contrast, LOESS does not require preset parameters, making it more flexible and suitable for in-depth analysis of long-term, nonlinear trend data [30].

Deep learning models, like Artificial Neural Networks (ANNs), are very useful for establishing regression models [116]. Large amounts of data provide ANNs with more information, helping the model to better learn patterns in the data, reduce the risk of overfitting, and improve accuracy and stability. Thus, deep learning models, including ANNs, are particularly suitable for large high-dimensional datasets [137,138]. Additionally, ANNs can automatically extract data features through deep structures, reducing the need for manual feature engineering [116,137,138,139]. This model is suitable for evaluating temporal trends; however, hyperparameter tuning is time-consuming, and its black-box nature often results in the poor interpretability of the results [116,139].

The RF regression model is also widely used. By analyzing the impact of multiple variables on outcomes and applying feature selection to reduce the adverse effects of high-dimensional data, this model is well suited for predicting continuous variables in complex datasets with missing data or nonlinear relationships [38,118,140]. While the RF regression model is practical for analyzing temporal trends, it may underperform when data is sparse [38,118,140,141].

The RF classification model, which is also based on the RF principle, is useful for regional epidemic risk assessment. By employing ensemble learning, bootstrap sampling, and random feature selection to construct decision trees, the RF classification model effectively handles high-dimensional data. However, this model is relatively inefficient, resource-intensive, and difficult to interpret [38,118,140,141].

The Bayesian model is another useful classification model that can quantify prediction uncertainty and probability. This model treats all parameters as random variables with certain distributions based on known information, thereby quantifying the credibility of each possible outcome [134]. By updating parameter classifications with new observational data, this model is particularly valuable in scenarios where sample data are limited, a common challenge in WBE. This model is also commonly used in regional classification and to enhance decision-making processes by providing a comprehensive view of uncertainty regarding parameters and predictions [134,142]. However, the accuracy of the Bayesian model relies on the choice of priors and assumptions. Incorrect information or assumptions can significantly impact the results [138,142,143]. Moreover, the Bayesian model struggles to draw effective inferences in high-dimensional spaces [142,143].

To optimize model performance, researchers often combine several models to leverage the strengths of each [36,144,145,146]. Hybrid models, which integrate multiple predictive techniques, can improve the robustness and accuracy of predictions. However, these models may introduce additional complexity, requiring more computational power and resources [30,37,38,39,41,108,115,116,117,118]. The exploration of more convenient and cost-effective models still requires more effort.

Despite their advantages, machine learning models must be validated and calibrated to ensure accuracy. Factors such as data quality, model selection, and computational constraints must be carefully managed to avoid biases and inaccuracies [30,36,37,38,39,41,108,115,116,117,118,141,144,145,146]. Therefore, it is important that models are carefully selected based on their predictive objectives.

### 4.3. Decision Making Based on Predictive Results

After proposing predictive results for models focused on epidemic temporal trends or regional risk stratification, feasible policies can be suggested to mitigate potential outbreaks. Planning models can then be used to evaluate the effectiveness of these policies [112,144,145,147]. Normative modeling approaches typically include optimization models, decision models, and simulation experiments.

Optimization models aim to maximize efficiency, particularly in resource allocation, allowing for parameter updates in response to new trends during the policy proposal phase and seeking to maximize efficiency under given conditions [147].

Once the optimized solutions are identified under different conditions, decision models are used to comprehensively evaluate various strategies and their potential outcomes. These models assist in selecting the most effective policies by comparing alternative monitoring strategies, assessing the risks associated with various viruses, and evaluating the long-term impact of the proposed interventions [145]. This step ensures that policymakers have a clear understanding of the trade-offs involved in each strategy [144].

Finally, simulation experiments can be conducted to comprehensively capture the dynamic changes within the system. These simulations reflect real-world randomness and variability, offering a more realistic representation of how the policies may perform [36,148]. Visualization tools are often employed to illustrate the changes within and between systems over time. Among the various simulation techniques, Monte Carlo simulations are widely used due to their effectiveness in assessing risks under uncertain conditions [149]. Based on simulation results, further policy adjustments or new policy proposals can be developed to enhance outbreak mitigation efforts.

## 5. Future Perspectives for WBE

WBE holds significant promise for monitoring viral infections and public health trends. However, several challenges must be addressed to fully realize its potential. This section outlines the key challenges in WBE and proposes future directions for improvement.

### 5.1. Improving WBE Accuracy

The primary value of WBE lies in its ability to provide timely, population-level health assessments. However, achieving consistent accuracy remains a challenge due to various factors, such as sample degradation, population characteristics, and variability in sewage systems [150,151]. To enhance the reliability of WBE, it is crucial to address these issues through improved sampling techniques, advanced detection methods, and refined data modeling.

One of the key challenges in WBE is the degradation of viral RNA during transport through wastewater systems. This degradation can lead to inaccuracies in concentration detection, ultimately affecting the reliability of WBE predictions [152]. To mitigate this issue, researchers have developed advanced sample stabilization techniques. For instance, the use of porous superabsorbent polymer (PSAP) beads has shown promise in significantly improving recovery efficiency [153]. Additionally, automated sampling and extraction methods have been introduced to minimize human error and ensure consistent sample quality [56,147,154,155].

Population characteristics also play a critical role in the accuracy of WBE. In communities with lower sewer connectivity, the representativeness of wastewater samples can be compromised, leading to biased results [156,157,158]. Rural areas or regions with decentralized sanitation systems may not be adequately represented in WBE studies, as their wastewater may not flow into centralized treatment plants. To address this, researchers must carefully select sampling sites that reflect the diversity of the population and consider alternative sampling strategies, such as targeting septic tanks or decentralized treatment systems in underserved areas [159].

Another factor affecting WBE accuracy is the variability in sewage systems, including differences in flow rates, pipe sizes, and wastewater retention times. These variables affect the concentration and stability of viral RNA in wastewater, complicating the acquisition of consistent measurements [160]. To improve accuracy, researchers are focusing on optimizing parameters such as sewer residence time (SRT) and dilution factors. By refining these parameters, WBE models can better account for the complex dynamics of wastewater systems and provide more reliable estimates of viral loads.

Environmental factors, such as temperature and dilution, also impact the accuracy of WBE. High wastewater temperatures (above 25 °C) or dilution with saline water can increase the sensitivity of viral RNA decay rates, leading to the potential underestimation of viral concentrations [51]. Similarly, rainwater dilution of domestic wastewater can affect the detection of bacterial pathogens, although it has less impact on viral pathogens. Therefore, it is essential to consider the decay behavior of specific pathogens under different environmental conditions when interpreting WBE data [151].

Flood-induced sewage overflows pose another challenge to WBE accuracy, particularly in older neighborhoods with combined sewer systems. During heavy rainfall, sewage overflows can lead to the spread of pathogens, complicating the interpretation of wastewater data [161]. To minimize this risk, local municipalities are encouraged to upgrade combined sewer systems to separate or partially separate systems, reducing the likelihood of pathogen spread during extreme weather events.

In addition to these technical challenges, the accuracy of WBE predictions depends on the robustness of data modeling [127]. Current models often struggle to capture the complex interplay between environmental variables, population dynamics, and viral transmission patterns. To address this, researchers are expected to develop more sophisticated models that integrate multiple data sources, including meteorological data, demographic information, and real-time wastewater measurements, which can be iteratively refined during implementation, allowing for more accurate and timely epidemic predictions [147].

### 5.2. Increasing Speed and Efficiency in WBE

WBE’s usefulness as a real-time monitoring tool depends heavily on the speed at which data can be collected and analyzed. Current methods, such as qPCR and sequencing, are time-consuming, and can delay public health interventions. To accelerate data processing, combined methods that integrate qPCR with sequencing or biosensor technologies should be prioritized. Additionally, accelerating model training and continuously refining these models during implementation will allow for quicker, more responsive analysis. This is especially critical during epidemic outbreaks, where timely data are essential for policy formulation and resource allocation [113]. However, current technologies mostly focus on the detection of single viruses, and the efficiency of detecting multiple viruses still needs improvement. Therefore, greater attention should be given to the differentiation of multiple viruses. Finally, mobile devices and internet-based tools can also help address the above issues [162].

### 5.3. Reducing Costs for Global WBE Accessibility

Cost remains a significant barrier to the widespread adoption of WBE, particularly in low-resource regions. The high costs associated with sophisticated equipment, highly trained personnel, and advanced detection technologies often limit WBE’s scalability in these regions. To make WBE more accessible globally, it is essential to develop cost-effective solutions that balance accuracy and affordability.

The development of low-cost detection platforms is one approach to reducing costs. Biosensors, for example, can detect viral RNA or proteins through biochemical reactions, making them suitable for use in resource-limited settings without the need for expensive equipment [99,100].

Optimizing sampling strategies can also contribute to cost reduction. In low- and middle-income countries, where sanitation facilities may be less developed, alternative sampling approaches are needed. For instance, in areas with decentralized sanitation systems, such as septic tanks or pit latrines, targeted sampling strategies can be employed to monitor specific populations without the need for extensive sewer network coverage [159]. However, challenges remain in defining the appropriate sampling scales and frequencies in these systems, particularly when factors such as toilet cleaning frequency or environmental decay rates are variable [163]. Developing standardized yet flexible sampling protocols for different sanitation systems can help reduce costs while ensuring data reliability.

### 5.4. Standardizing Global WBE Practices

Currently, there are no universally accepted standards for pathogen detection and reporting in WBE, leading to discrepancies in data across regions [164]. This lack of standardization hampers global comparisons and reduces the overall utility of WBE as a public health tool [145,165,166]. Establishing global standards for sample collection, detection methods, and data reporting is essential to ensure consistency, comparability, and reliability in WBE practices [68].

Publishing direct measurement data—such as gene copy numbers per liter of wastewater or per gram of sludge, alongside recovery efficiency and other quality control metrics—can improve cross-study comparability [152]. Researchers have also called for the development of universally accessible proxy viruses to standardize recovery rates and enhance comparability across laboratories [167]. Additionally, safety is also paramount in WBE sampling to protect researchers from exposure risks. Since viruses in wastewater can remain infectious, sampling personnel must follow strict safety protocols. Heat inactivation of viral standards (e.g., Vesicular Stomatitis Virus, or VSV) should be employed to minimize the risk of exposure [49].

The establishment of national international monitoring networks is a necessary step toward global standardization. For instance, the U.S. Centers for Disease Control and Prevention (CDC) established the National Wastewater Surveillance System (NWSS) in September 2020, which rapidly expanded to over 1500 sampling sites by December 2022, covering approximately 47% of the U.S. population [168]. The NWSS focuses on the impact of sampling techniques, population distribution, social vulnerability index, and detection methods, aiming to build a flexible national monitoring system. However, challenges remain, such as obtaining complete geographic data for sewer boundaries and ensuring consistent sampling frequency across sites [168]. Similarly, China has begun to build a nationwide unified sewage COVID-19 virus monitoring information system, with more than 3000 monitoring points expected to be covered by 2025 [169]. Regional monitoring systems, such as the Guangdong Provincial Sewage Information Analysis and Early Warning Key Laboratory, have also been established to address local needs. However, balancing temporal and spatial factors remains challenging, particularly in regions with significant seasonal variations or disparities in public health policies [170,171].

### 5.5. Ethical and Privacy Considerations in WBE

Although WBE monitors population health trends rather than individuals, it still raises concerns about privacy and consent [172]. Collecting wastewater data without explicit consent can lead to ethical dilemmas regarding data ownership and public health surveillance boundaries. Government authorities and public health departments can use wastewater monitoring for community health surveillance, collecting relevant data through institutions such as disease control centers and statistical agencies to provide data support for epidemic control and policy decisions. In addition, hospitals and research institutions, as collectors and processors, assist government departments, but they do not hold the legal responsibility for this information as individuals [173,174,175]. To mitigate these concerns, clear guidelines must be developed to protect individual privacy while allowing for the collection of crucial public health data. Engaging communities and policymakers in discussions about WBE’s benefits and limitations will help establish ethical frameworks that ensure the responsible use of wastewater data.

### 5.6. Expanding WBE Targets

As WBE evolves, future efforts should focus on expanding its scope beyond pathogen detection. By targeting a broader range of biomarkers, WBE can provide a more comprehensive view of public health risks and enhance its utility as a proactive surveillance tool. Emerging markers in WBE include the detection non-communicable diseases and other health indicators. For example, changes in the microbial community composition of wastewater can reflect shifts in population health, such as the prevalence of metabolic disorders or chronic diseases [176].

Additionally, WBE holds significant potential for tracking chemical markers, such as pharmaceuticals and environmental pollutants. These substances not only reflect population-level exposure to pollutants but also serve as indirect indicators of public health status [177,178].

Another promising direction is the application of WBE to monitor antibiotic resistance genes (ARGs) and other emerging biomarkers. The spread of antimicrobial resistance is a growing global health concern, and WBE offers a unique opportunity to track ARGs in wastewater, providing insights into the prevalence and transmission of resistant bacteria within communities [177,178,179,180,181,182,183,184,185].

While this review does not delve into the technical and interpretive challenges of expanding WBE targets, it is crucial to emphasize that detecting and analyzing the aforementioned biomarkers represents a pivotal step toward refining public health interventions. To fully realize WBE’s transformative potential, future research can systematically explore novel applications, diversify biomarker panels, and integrate multiomics approaches to strengthen its role as a predictive and preventive public health tool.

## 6. Conclusions

WBE has shown great potential to revolutionize public health surveillance by offering a non-invasive and scalable approach to monitor population-level health dynamics. This article systematically reviewed the core processes of WBE, from sampling and pathogen detection to epidemic forecast modeling, highlighted its role in early outbreak warnings, resource allocation, and public health decision making. However, further efforts are needed to improve accuracy, efficiency, and cost-effectiveness, as well as to establish universal standards for data collection and analysis. Furthermore, ethical considerations, such as privacy concerns, also need to be addressed. In the future, WBE has the potential to expand beyond pathogen detection, including the monitoring of antibiotic resistance genes, chemical markers, and other emerging health indicators. By integrating artificial intelligence and real-time monitoring systems, its predictive power can be further enhanced. With sustained innovation and interdisciplinary collaboration, WBE can play an important role in preventing future pandemics, managing epidemic outbreaks, and improving overall population health globally.

## Figures and Tables

**Table 1:** Comparison of different viral detection methods.

Method	qPCR	Amplicon Sequencing	Metagenomic Sequencing	Biosensors
Principle	External amplification of DNA	DNA/RNA sequencing	DNA/RNA sequencing	Specific receptors emit a clear response signal
Target	Specific region of viral genome	Specific region of viral genome	Whole viral genome	Specific DNA/RNA/protein
Time	Short	Long	Long	Short
Precision	High	High	High	Amplification-based: low
Cost	Low	Low	High	Low
VOC discoverable	No	Only when mutation sites are in the amplified region	Yes	No

**Table 2:** Comparison of different modeling methods in WBE.

Method	Applicable Conditions	Advantages	Limitations
SMA	Smoothing short-term date	Simple, stable, and easy to implement	Slow response to emergencies
LOESS	Long-term and nonlinear data	No need to set parameters in advance	High computational complexity
ANNs	Large amounts of data and time-consuming process	Extract data features through deep structures	Time-consuming and poor interpretability of the results
Random Forest	High-dimensional data with missing data or nonlinear relationships	Prevent overfitting	Relatively inefficient, resource-intensive, and difficult to interpret
Bayesian Model	Sample data are insufficient in low-dimensional spaces	Quantify the credibility of each possible outcome	Relies on the choice of priors and assumptions

## Data Availability

No new data were created or analyzed in this study.

## Abbreviations

The following abbreviations are used in this manuscript:

Abbreviation	Full Form
WBE	Wastewater-based epidemiology
LC-MS	Liquid chromatography-mass spectrometry
qPCR	Quantitative polymerase chain reaction
GIS	Geographic information system
VOCs	Variants of concerns
NGS	Next-generation sequencing
ddPCR	Droplet digital polymerase chain reaction
MST	Microbial source tracking
HTS	High-throughput sequencing
RT-LAMP	Reverse transcription loop-mediated isothermal amplification
NASBA	Nucleic acid sequence-based amplification
FET	Field-effect transistors
mHealth	Mobile health
SVR	Support vector regression
ANN	Artificial neural network
RF	Random forest
SVM	Support vector machine
SMA	Simple moving average
LOESS	Locally weighted regression
PSAP	Porous superabsorbent polymer
SRT	Sewer residence time
VSV	Vesicular stomatitis virus
CDC	Center for Disease Control and Prevention
NWSS	National Wastewater Surveillance System
ARGs	Antibiotic resistance genes
